# Supplementary material for: Latent transition analysis for longitudinal studies of post-acute infection syndromes
Source: Nat Commun. 2026 Feb 10;17:2557. doi: 10.1038/s41467-026-68650-7 (PMC13000239; doi:10.1038/s41467-026-68650-7)
Supplement: Supplementary file 1 — Supplementary Information [file 41467_2026_68650_MOESM1_ESM.pdf]

Supplementary Information of Latent transition analysis for longitudinal studies of post-acute infection syndromes

This section is a supplementary to the paper, “Latent transition analysis for longitudinal studies of post-acute infection syndromes” and contains the list of consortia members, further mathematical derivations of the model, supplementary tables, as well as supplementary figures to provide added details presented in the paper.

1 Supplementary Notes

This section serves as a supplement to the Methods section of the paper.

1.1 Derivations

In this section we detail the model and its corresponding components used within the paper.

1.1.1 Notation

The three key components of interest in defining the HMM model are as follows.

Let π_i be the probability of being in a state at the initial timepoint, $t = 0$ such that $\pi_i = P(S_i^{t=0} = 1)$. We wish to make the initial state distribution conditioned on a patient's covariates, $\mathbf{C}^{initial}$ by parameterising via a multinomial function again with $\beta_{hj}^{initial} \in \mathbb{R}^{2\times(N-1)}$ and the first state chosen as the reference. However, noting that all states except one have a corresponding parameter set β, the total number of parameters to define the initial distribution is $(N-1)(K^{initial}+1)$. In order to reduce the complexity of the model while still estimating the general effect of a patient's characteristics, we collapse the covariate vector to a scalar value, $r^{initial}$ by taking the inner product with parameter vector, $\boldsymbol{\rho}^{initial}$, such that $r^{initial} = \boldsymbol{\rho}^{initial} \cdot \mathbf{C}^{initial}$. This value is then used as the single aggregated independent variable within the multinomial function,

$$P\left(S_i^{t=0} = 1 \mid \mathbf{C}^{initial}\right) \equiv \pi_i = \begin{cases} \dfrac{1}{1 + \sum\limits_{\substack{j=1 \\ j\neq i}}^{N} \exp\left(\beta_{1j}^{initial} + \beta_{2j}^{initial}\, r^{initial}\right)} & \text{if } i = 1, \\[2ex] \dfrac{\exp\left(\beta_{1j}^{initial} + \beta_{2j} r^{initial}\right)}{1 + \sum\limits_{\substack{j=1 \\ j\neq i}}^{N} \exp\left(\beta_{1j}^{initial} + \beta_{2j}^{initial}\, r^{initial}\right)} & \text{if } i \neq 1, \end{cases} \tag{1}$$

and the number of parameters is $2(N-1) + K^{initial}$ and notably does not scale multiplicatively with the number of states and covariates within the model.

Let a be the matrix of transition probabilities between states, such that $a_{ij} = P(S_j^t = 1|S_i^{t-1} = 1) = P(S_j^t|S_i^{t-1})$ is the probability of moving from the $i-$th state to $j-$th state in a single timestep. We note that these probabilities remain independent of the timepoint t. No restrictions are placed on the transition probabilities between states, so the latent states are fully connected and there is a non-zero probability of jumping from any given state to any other state within a single timestep. This importantly includes the possibility to remain within a state. Similarly to how we parameterised the initial state distribution, the probability of moving into a j-th state from the i-th state is described by the following function for each state,

$$P\left(S_j^t = 1 \mid S_i^{t-1} = 1,\ \mathbf{C}^{trans}\right) \equiv a_{ij} = \begin{cases} \dfrac{1}{1 + \sum\limits_{\substack{j=1 \\ j\neq i}}^{N} \exp\left(\beta_{1ij}^{trans} + \beta_{2ij}^{trans}\, r^{trans}\right)} & \text{if } i = j, \\[2em] \dfrac{\exp\left(\beta_{1ij}^{trans} + \beta_{2ij} r^{trans}\right)}{1 + \sum\limits_{\substack{j=1 \\ j\neq i}}^{N} \exp\left(\beta_{1ij}^{trans} + \beta_{2ij}^{trans}\, r^{trans}\right)} & \text{if } i \neq j, \end{cases} \tag{2}$$

where $r^{trans} = \boldsymbol{\rho}^{trans} \cdot \mathbf{C}^{trans}$ reduces the set of covariates. This ensures that transition matrix a is row-stochastic and dependant on a patient's covariates. The total number of parameters now becomes $2N(N-1) + K^{trans}$, thus the computational complexity does not increase greatly when more transition covariates are added to the model.

Lastly, each state has an associated probability of manifesting the l-th component of the observation vector, $\mathbf{X}^t$, which depends only on being in the state at the given timepoint t. So, we define the probability of manifesting an observation X_l^t from the i-th state as

$$P\left(X_l^t \mid S_i^t = 1\right) \equiv c_i\left(X_l\right),$$

noting that the relation between the observation and state is independent of the timepoint. If the observation is binary, such as the occurrence of symptoms like anosmia, we model it as being drawn from a Bernoulli distribution with probability b_{il}. In the case of continuous observations like the HRQoL scores, we assume it to be drawn from a Gaussian distribution with associated mean μ_{il} and standard deviation, σ_{il}. Thus we have,

$$\begin{aligned} X_l = 1 \mid S_i = 1 &\sim \text{Bernoulli}(b_{il}) \quad \text{if observation } X_l \text{ is binary,} \\ X_l = x \mid S_i = 1 &\sim \text{Normal}\left(\mu_{il}, \sigma_{il}\right) \quad \text{if observation } X_l \text{ is continuous.} \end{aligned}$$

Because $b_{ij}, \mu_{il} \in [0,1]$ as the scores have been normalised and $\sigma_{il} \in [0, \infty)$, we express these quantities as,

$$b_{il} = \text{logistic}(p_{il}) = \frac{1}{1 + e^{-p_{il}}}; \quad \mu_{il} = \text{logistic}(u_{il}); \quad \sigma_{il} = e^{v_{il}}.$$

In order to calculate corresponding confidence intervals at a specified significance level, α, we simply

take the inverse functions,

$$\begin{aligned} CI_{\hat{\mu}} &: \left[\text{logistic}\big(\hat{u} - z_{1-\alpha/2}s_{\hat{u}}\big),\ \text{logistic}\big(\hat{u} + z_{1-\alpha/2}s_{\hat{u}}\big)\right], \\ CI_{\hat{\sigma}} &: \left[e^{\hat{v} - z_{1-\alpha/2}s_{\hat{v}}},\ e^{\hat{v} + z_{1-\alpha/2}s_{\hat{v}}}\right], \end{aligned}$$

where $z_{1-\alpha/2}$ is the z-score.

So, the complete set of parameters is now $\theta = \{\boldsymbol{\rho}^{initial}, \beta^{initial}, \boldsymbol{\rho}^{trans}, \beta^{trans}, p_{il}, u_{il}, v_{il}\}$.

1.1.2 Forward Algorithm for HMM Likelihood Computation

Rather than deriving the explicit update step for our model, we derive the likelihood function and then utilise a BFGS to fit the parameters which give the largest likelihood. To do this efficiently, we take the forward section of the forward-backward algorithm to construct the likelihood using dynamic programming.

We denote the forward path probability of being in the j-th state at time t, with all observations up until that time as,

$$\alpha_t(j) \equiv P\left(\mathbf{X}^1, \mathbf{X}^2, \ldots, \mathbf{X}^t, S_j^t | \theta\right).$$

Considering the $i-$th state, S_i, we demonstrate the case where there is a vector of observations, $\mathbf{X}$. We note $b_i\left(X_l^t\right)$ is the probability of the l-th observation at time t given the $i-th$ state. For the initialisation then,

$$\begin{aligned} \alpha_1(j) &= P\left(S_j^1\right) P\left(\mathbf{X}^1 | S_j^1\right) \\ &= \pi_j P\left(X_1^1, X_2^1, \ldots, X_L^1 \mid S_j^1\right) \\ &= \pi_j P\left(X_1^1 \mid S_j^1\right) P\left(X_2^1 \mid S_j^1\right) \ldots P\left(X_L^1 \mid S_j^1\right) \\ &= \pi_j c_j\left(X_1^1\right) c_j\left(X_2^1\right) \ldots c_j\left(X_L^1\right) \\ &= \pi_j \prod_{l=1}^{L} c_j\left(X_l^1\right), \end{aligned}$$

as we consider observations of different symptoms conditionally independent of each other. Similarly, the iterative rule of α_t is changed to,

$$\alpha_t(j) = \sum_{i=1}^{N} \alpha_{t-1}(i) a_{ij} \prod_{l=1}^{L} c_j\left(X_l^t\right).$$

Terminating the iteration at the last timepoint, $t = T$ and summing across the states thus yields the likelihood of sample sequence,

$$\sum_{j=1}^{N} \alpha_T(j) = P\left(\mathbf{X}^1, \mathbf{X}^2, \ldots, \mathbf{X}^T | \theta\right).$$

We reformulate the algorithm now such that the small unit is in log space. From the likelihood, the algorithm for computing the full likelihood of a dataset, $\mathcal{D}$ consisting of D patients and given

model parameters, θ is

$$\begin{aligned}\mathcal{L}\left(\theta \mid \mathcal{D}\right) &= \log \prod_{d=1}^{D} \sum_{j=1}^{N} \alpha_T^d(j) \\ &= \sum_{d=1}^{D} \log \sum_{j=1}^{N} e^{\log \alpha_T^d(j)}.\end{aligned}$$

Thus, for a given sample, we have the initialisation step,

$$\log \alpha_1(j) = \log \pi_j + \log c_j\left(X_1^1\right) + \log c_j\left(X_2^1\right) + \cdots + \log c_j\left(X_L^1\right),$$

and for the general update rule,

$$\log \alpha_t(j) = \sum_{l=1}^{L} \log c_j\left(X_l^t\right) + \log\left(\sum_{i=1}^{N} e^{\log \alpha_{t-1}(i) + \log a_{ij}}\right).$$

1.1.3 Probability of Latent State given Multiple Observations (Filtering)

We may also calculate the distribution of states given the previous observation in order to update model predictions. Noting that,

$$\begin{aligned}P\left(S_i^t \middle| \mathbf{X}^{1:t}, \theta\right) &= \frac{P\left(S_i^t,\, \mathbf{X}^{1:t} \,|\theta\right)}{P\left(\mathbf{X}^{1:t} \mid \theta\right)} = \frac{P\left(S_i^t,\, \mathbf{X}^{1:t} \,|\theta\right)}{\sum_{j=1}^{N} P\left(S_j^t, \mathbf{X}^{1:t} \mid \theta\right)} \\ &= \frac{\alpha_t(i)}{\sum_{j=1}^{N} \alpha_t(j)},\end{aligned}$$

using the definition of conditional probability and seeing that the total probability of the observed data comes from summing the joint probabilities from each state.

We may also then calculate the distribution of future observations given all observations up to a given time t, $P\left(\mathbf{X}^{1:t+1} \middle| \mathbf{X}^{1:t}, \theta\right)$.

$$
\begin{aligned}
P\left(\mathbf{X}^{1:t+1}|\mathbf{X}^{1:t},\theta\right) &= \sum_{j=1}^{N} P\left(\mathbf{X}^{1:t+1}, S_j^{t+1}|\mathbf{X}^{1:t},\theta\right) \\
&= \sum_{j=1}^{N} P\left(\mathbf{X}^{1:t+1}|S_j^{t+1},\mathbf{X}^{1:t},\theta\right) P\left(S_j^{t+1}|\mathbf{X}^{1:t},\theta\right) \\
&= \sum_{j=1}^{N} c_j\left(\mathbf{X}^{t+1}\right) \sum_{i=1}^{N} P\left(S_j^{t+1}, S_i^{t}|\mathbf{X}^{1:t},\theta\right) \\
&= \sum_{j=1}^{N} c_j\left(\mathbf{X}^{t+1}\right) \sum_{i=1}^{N} P\left(S_j^{t+1}|S_i^{t},\mathbf{X}^{1:t},\theta\right) P\left(S_i^{t}|\mathbf{X}^{1:t}\right) \\
&= \sum_{j=1}^{N} c_j\left(\mathbf{X}^{t+1}\right) \sum_{i=1}^{N} a_{ij} P\left(S_i^{t}|\mathbf{X}^{1:t}\right),
\end{aligned}
$$

using the chain rule and definition of the emission and transition probabilities. Thus, we may compute the distribution of observations by updating the state distribution at that timepoint, given observations at the previous timepoints.

1.1.4 Handling Missing Observations using Full-Information Maximum Likelihood

Consider the case where an observation, X_m^t is missing. We can marginalise out the effect by

$$
\begin{aligned}
\alpha_t(j) &= \int P\left(\mathbf{X}^{1:t}, S_j^t\right) dX_m^t \\
&= \int P\left(\mathbf{X}^{1:t-1}, X_1^t, \ldots, X_L^t, S_j^t\right) dX_m^t \\
&= \int \prod_{l=1}^{L} c_j\left(X_l^t\right) dX_m^t \sum_{i=1}^{N} \alpha_{t-1}(i) a_{ij} \\
&= \int c_j\left(X_m^t\right) dX_m^t \prod_{l=1;\, l\neq m}^{L} c_j\left(X_l^t\right) \sum_{i=1}^{N} \alpha_{t-1}(i) a_{ij} \\
&= \prod_{l=1;\, l\neq m}^{L} c_j\left(X_l^t\right) \sum_{i=1}^{N} \alpha_{t-1}(i) a_{ij},
\end{aligned}
$$

as we note that $\int c_j\left(X_m^t\right) dX_m^t = 1$ by definition. Thus the forward update in the case of a missing observation is equivalent to ignoring the observation in regular forward update. Rather than computing the integral directly at each step, we can simply redefine,

$$
\tilde{c}_j(X_l^t) = \begin{cases} c_j(X_l^t), & \text{if } X_l^t \text{ is observed}, \\ 1, & \text{if } X_l^t \text{ is missing}. \end{cases}
$$

Thus, with a minor adjustment, we may compute the likelihood efficiently with missing observations, rather than relying on imputation based methods, which may be prone to bias estimation results.

1.1.5 Steady State

The steady state distribution, ν of a row-stochastic transition probability matrix, A is defined as $\nu = A\nu$. That is, the steady state distribution remains unchanged after transitioning from one timepoint to another. Noting that when our Markov chain is aperiodic and irreducible then there exists a steady distribution, with the first eigenvector of A equal to ν.

2 Supplementary Tables

2.1 Estimated Model Parameters

	States						
Symptoms	Acute Respiratory	Acute Moderate	Healthy	Sensorial PCC	Respiratory PCC	Fatigue PCC	Severe Symptoms
**Ageusia**	0.0655	0.0830	0.0121	0.8149	0.0031	0.0421	0.9508
**Anosmia**	0.0365	0.0390	0.0227	0.8940	0.0045	0.0364	0.9827
**Arthralgia**	0.0118	0.4938	0.0460	0.0387	0.0874	0.5578	0.4376
**Cough**	0.7399	0.7742	0.0496	0.1127	0.1031	0.2972	0.7100
**Dyspnea**	0.5656	0.6005	0.0638	0.1225	0.3050	0.5880	0.6455
**Fatigue**	0.5419	0.8149	0.1409	0.3136	0.4292	0.8426	0.8646
**Headache**	0.1570	0.4837	0.0420	0.1117	0.0463	0.3180	0.5641
**Memory Loss**	0.0168	0.0616	0.0810	0.1274	0.1523	0.4238	0.2475
**Mylagia**	0.0758	0.9127	0.0427	0.0291	0.0749	0.5954	0.6566

Supplementary Table 1: **Emission Probabilities of 7 State Model.** Probability (%) of observing each symptom given the latent state.

Physical Component Estimates				
State	Mean ($\hat{\mu}$)	95% CI	SD ($\hat{\sigma}$)	95% CI
**Acute Respiratory**	0.1408	[0.0, 1.0]	0.1407	[0.0, 5.5472e7]
**Acute Moderate**	0.1409	[0.0, 1.0]	0.1411	[0.0, 7.1134e7]
**Healthy**	0.5431	[0.5402, 0.546]	0.0530	[0.0509, 0.0552]
**Sensorial PCC**	0.4594	[0.445, 0.4738]	0.1183	[0.1091, 0.1281]
**Respiratory PCC**	0.4686	[0.4613, 0.4759]	0.1104	[0.1057, 0.1154]
**Fatigue PCC**	0.3939	[0.3826, 0.4052]	0.1256	[0.1191, 0.1324]
**Severe Symptoms**	0.4334	[0.4116, 0.4555]	0.1160	[0.1028, 0.1309]

Supplementary Table 2: **Estimated Parameters of Gaussian Distribution for Physical Scores.** Parameter Estimates of Means and Standard Deviation of Normal Distribution per state for Physical Component Scores.

Mental Component Estimates				
State	Mean ($\hat{\mu}$)	95% CI	SD ($\hat{\sigma}$)	95% CI
**Acute Respiratory**	0.1408	[0.0, 1.0]	0.1411	[0.0, 4.9319e7]
**Acute Moderate**	0.1410	[0.0, 1.0]	0.1399	[0.0, 1.9695e8]
**Healthy**	0.5762	[0.5746, 0.5777]	0.0269	[0.0256, 0.0282]
**Sensorial PCC**	0.5438	[0.5332, 0.5542]	0.0793	[0.072, 0.0873]
**Respiratory PCC**	0.4772	[0.4704, 0.4841]	0.0959	[0.092, 0.0999]
**Fatigue PCC**	0.4022	[0.3911, 0.4135]	0.1000	[0.0937, 0.1068]
**Severe Symptoms**	0.4235	[0.4023, 0.445]	0.1098	[0.0969, 0.1243]

Supplementary Table 3: **Estimated Parameters of Gaussian Distribution for Mental Scores.** Parameter Estimates of Means and Standard Deviation of Normal Distribution per state for Mental Component Scores.

Initial State Probability Covariates ($\hat{\boldsymbol{\rho}}_{initial}$)			
**Covariate**	**Estimate**	**95% CI**	**p-value**
Female	-0.1950	[-1.3689, 0.9788]	0.7447
Age 31–40	0.1700	[-0.8917, 1.2317]	0.7537
Age 41–60	0.1515	[-0.7977, 1.1007]	0.7544
Age $>$ 60	0.5079	[-2.5804, 3.5962]	0.7472
Second Wave	-0.4266	[-2.9959, 2.1426]	0.7448
Third Wave	-0.2336	[-1.6426, 1.1754]	0.7452
Fourth Wave	-0.0273	[-0.2321, 0.1774]	0.7937
Post–Fourth Wave	0.5396	[-2.7197, 3.7989]	0.7456
Corticosteroids	0.1963	[-0.9848, 1.3773]	0.7446
Respiratory Disease	0.0468	[-0.2539, 0.3475]	0.7604
Invasive ventilation	0.3477	[-1.7609, 2.4563]	0.7466
Non-invasive ventilation	0.3228	[-1.6329, 2.2785]	0.7463

Supplementary Table 4: **Parameter Estimates of $\hat{\boldsymbol{\rho}}_{initial}$.** A Wald test was performed for each parameter where all p-values were two-sided. No adjustment for multiple comparisons was applied. The 95% confidence bands were computed from the parameter variance–covariance matrix.

Transition State Probability Covariates ($\hat{\boldsymbol{\rho}}_{trans}$).			
**Covariate**	**Estimate**	**95% CI**	**p-value**
Female	0.4104	[0.0522, 0.7686]	0.0247
Age 31–40	0.2322	[-0.0473, 0.5117]	0.1034
Age 41–60	0.4042	[0.0244, 0.7841]	0.0370
Age > 60	0.5022	[0.0455, 0.9589]	0.0312
Second Wave	-0.1846	[-0.3785, 0.0094]	0.0621
Third Wave	-0.3460	[-0.6623, -0.0298]	0.0320
Fourth Wave	-0.3528	[-0.6848, -0.0208]	0.0373
Post–Fourth Wave	-0.0912	[-0.2441, 0.0617]	0.2422
Corticosteroids	0.2475	[0.0185, 0.4764]	0.0341
Respiratory Disease	0.3302	[0.0373, 0.6232]	0.0271
Invasive ventilation	0.0796	[-0.0232, 0.1823]	0.1290
Non-invasive ventilation	0.1831	[-0.0077, 0.3738]	0.0599

Supplementary Table 5: **Parameter Estimates of $\hat{\boldsymbol{\rho}}_{trans}$. A Wald test was performed for each parameter where all p-values were two-sided. No adjustment for multiple comparisons was applied. The 95% confidence bands were computed from the parameter variance–covariance matrix.**

2.2 Mean State Probabilities (%)

State	Month 0	Month 6	Month 12	Month 18	Month 24
**Acute Respiratory**	51.17	0.00	0.00	0.00	0.00
**Acute Moderate**	21.37	0.00	0.00	0.00	0.00
**Healthy**	1.94	32.56	35.44	37.52	39.15
**Sensorial PCC**	3.56	5.88	5.99	5.97	5.97
**Respiratory PCC**	4.78	41.53	39.02	37.02	35.53
**Fatigue PCC**	0.58	16.18	17.03	17.15	17.03
**Severe Symptoms**	16.61	3.85	2.52	2.34	2.32

Supplementary Table 6: **Mean State Probabilities (%).** The average probability of state occurrence at each timepoint.

3 Supplementary Figures

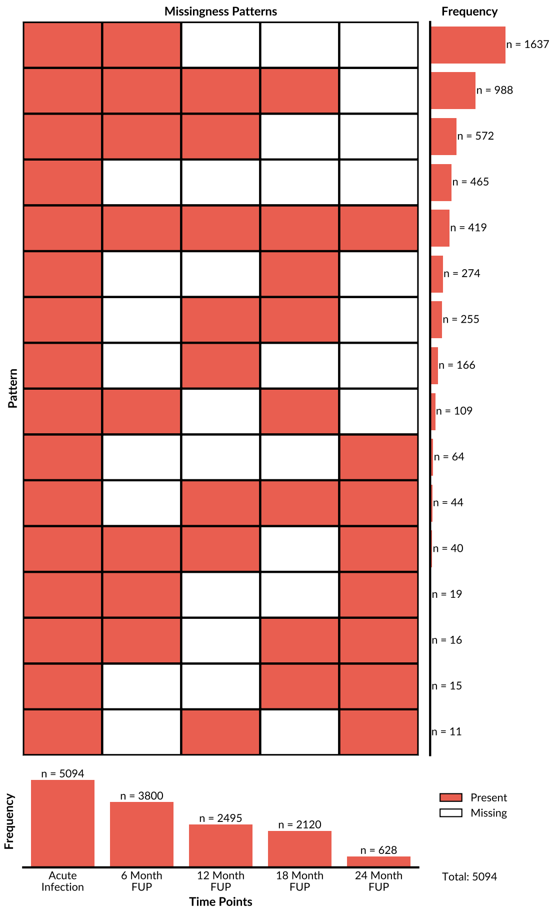

Supplementary Figure 1: **Missingness Pattern of the ORCHESTRA Dataset.** Missingness pattern of the follow-up across the whole cohort study.

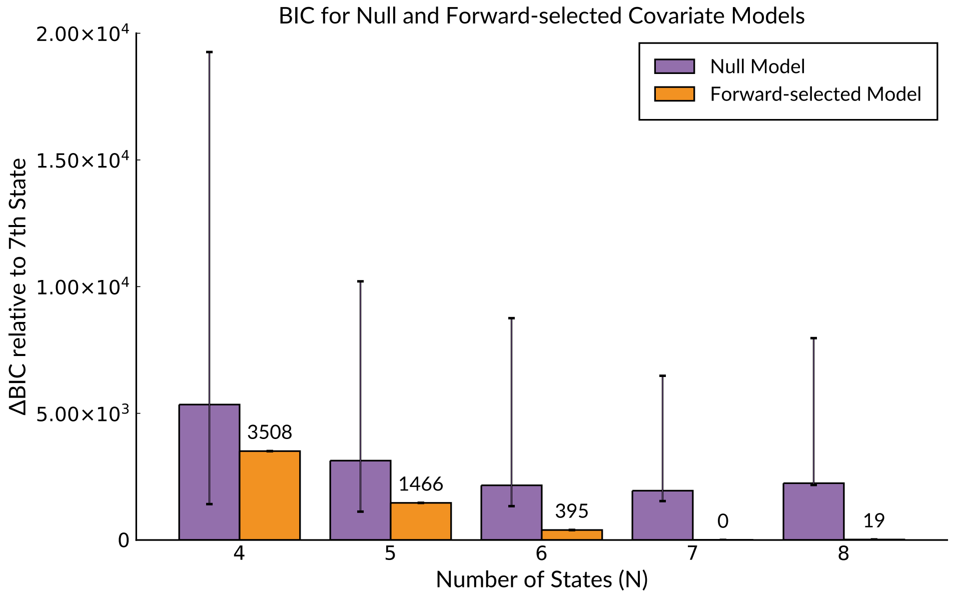

Supplementary Figure 2: **BIC Comparison across Models.** Comparing BIC Values between Null and Forward-selected Covariate Models against the 7 State Covariate Model. Error bars indicate highest and lowest values across 20 multistart runs.

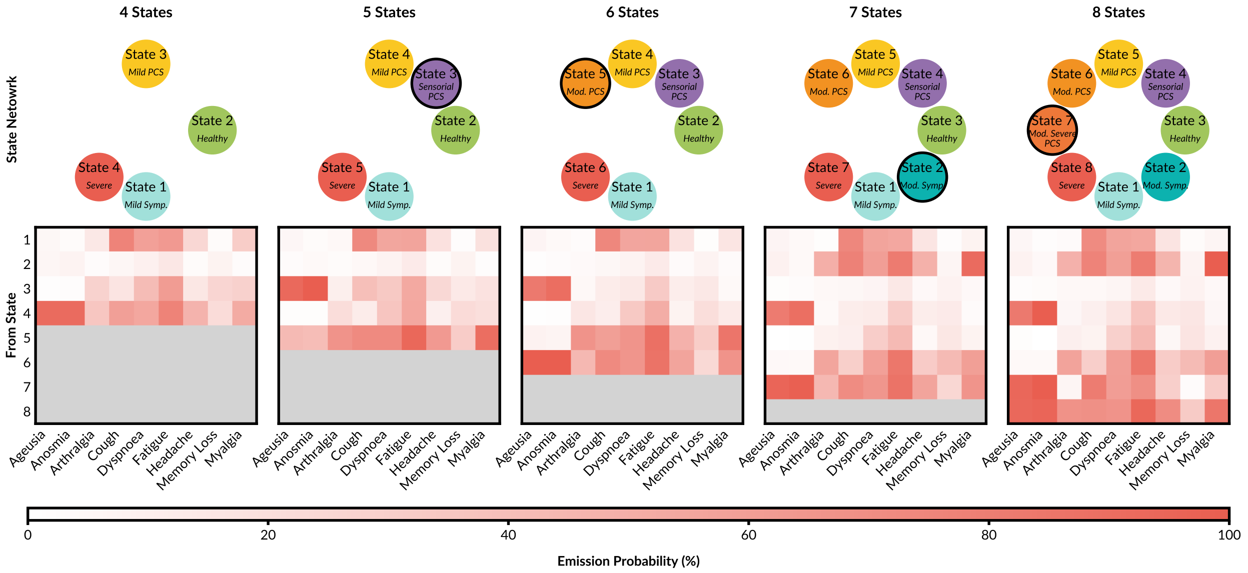

Supplementary Figure 3: **Comparison of Emission Matrices and Identified States.** Emission matrices of Forward-selected Model for varying number of states (N). Newly added states are highlighted with a black border.

Forward Selection Process across Model Sizes (4 to 8 States)

Covariate	N = 4	N = 5	N = 6	N = 7	N = 8
Hospital Admission					
Age	0	0	0	0	0
Sex	0	0	0	0	0
Infection Wave	2	2	1	2	
Chronic Respiratory Disease	1	1		3	2
Oxygen Therapy	3			1	3
Corticosteroids		4	2	4	
Monoclonal Antibodies		3			1
Immunomodulators		5			
Previous Smoker					
Antivirals (Remdesivir)					
Vaccination before Acute Infection					
Model BIC	59784.15	57741.57	56670.70	56276.02	56295.32
Difference between Model BIC and N = 7 BIC (ΔBIC)	+3508.13	+1465.55	+394.68	+0.00	+19.30

Supplementary Figure 4: **Selection of Covariates across Model Sizes.** The enhanced table displays which covariates were selected during the forward model selection process for model sizes $N = 4$ to $N = 8$. The integer value within the cell indicates at which selection round was the covariates chosen, with 1 being the first covariate chosen. A grey strike indicates the covariate was not chosen. Hospitalisation was excluded from the list of possible covariates, while age and sex were always included in every model. The 7 state model was found to have the lowest BIC value.

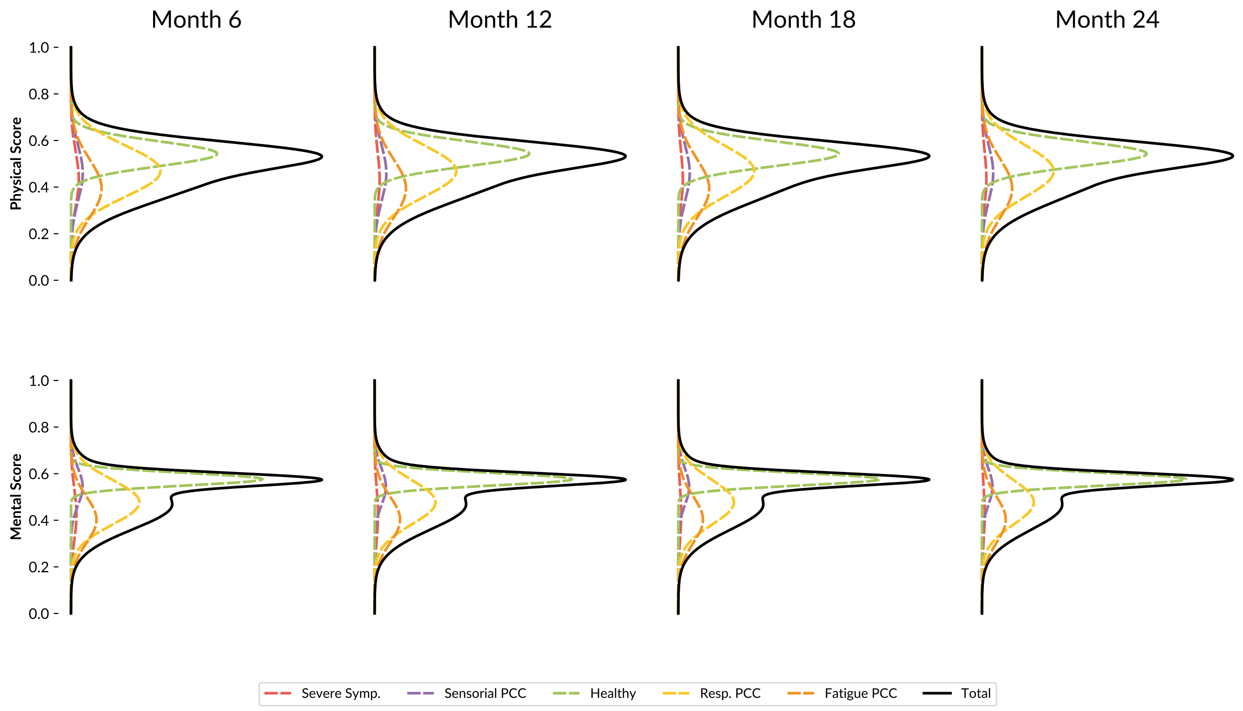

Supplementary Figure 5: **State Contribution of HRQoL Scores.** Population-level predictions of SF-36 Physical and Mental HRQoL scores.

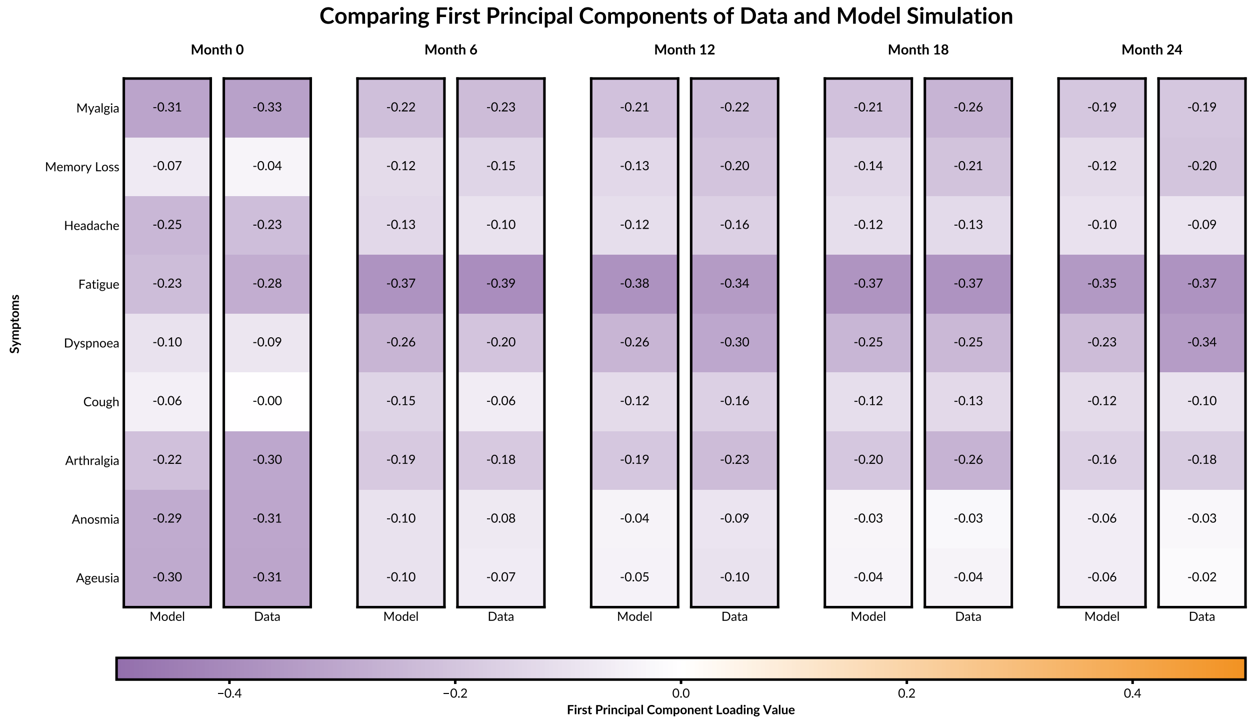

Supplementary Figure 6: **First Principal Component Comparison of Data and Model Simulation.** Heatmaps comparing the loading values of the first component for the dataset and model simulation (average across 1000 simulations).

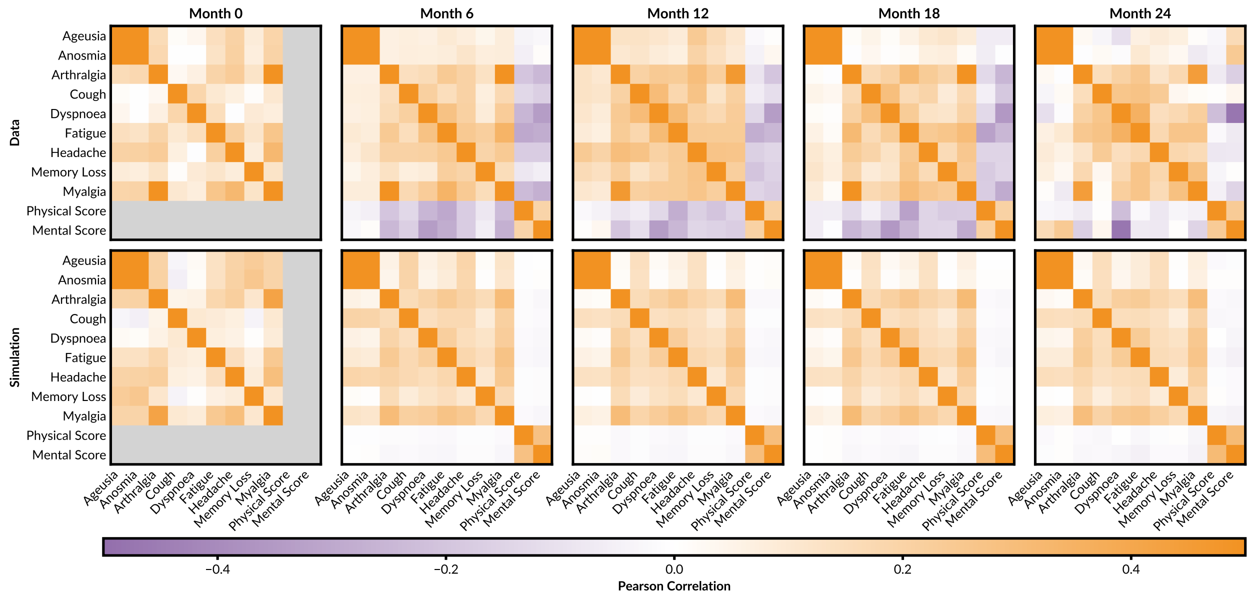

Supplementary Figure 7: **Correlation Matrix Comparison of Data and Model Simulation.** Heatmaps comparing the Pearson Correlation values of the nine symptoms and HRQoL scores for the dataset and model simulation (average across 1000 simulations).

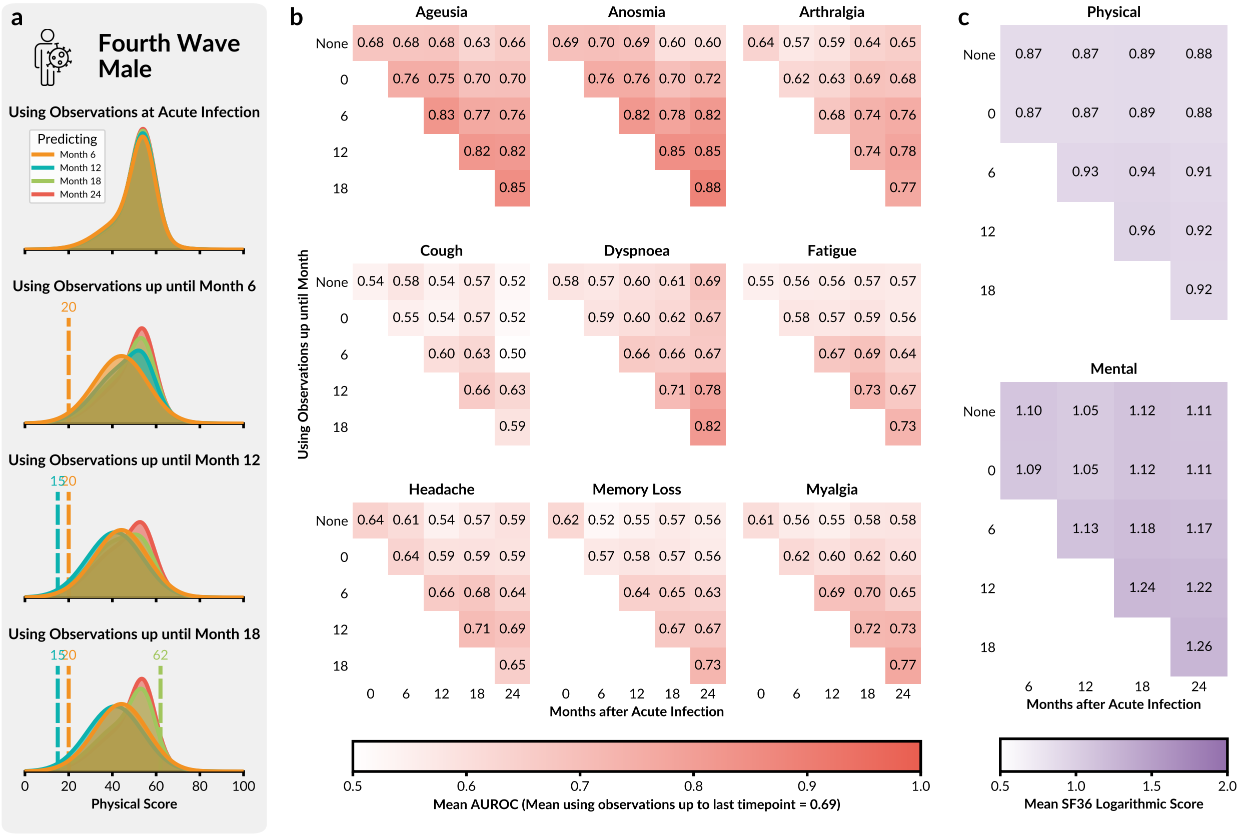

Supplementary Figure 8: **True AUROC and Mean Log Values.** AUROC and Mean Log Values of Symptoms and SF-36 Scores respectively.

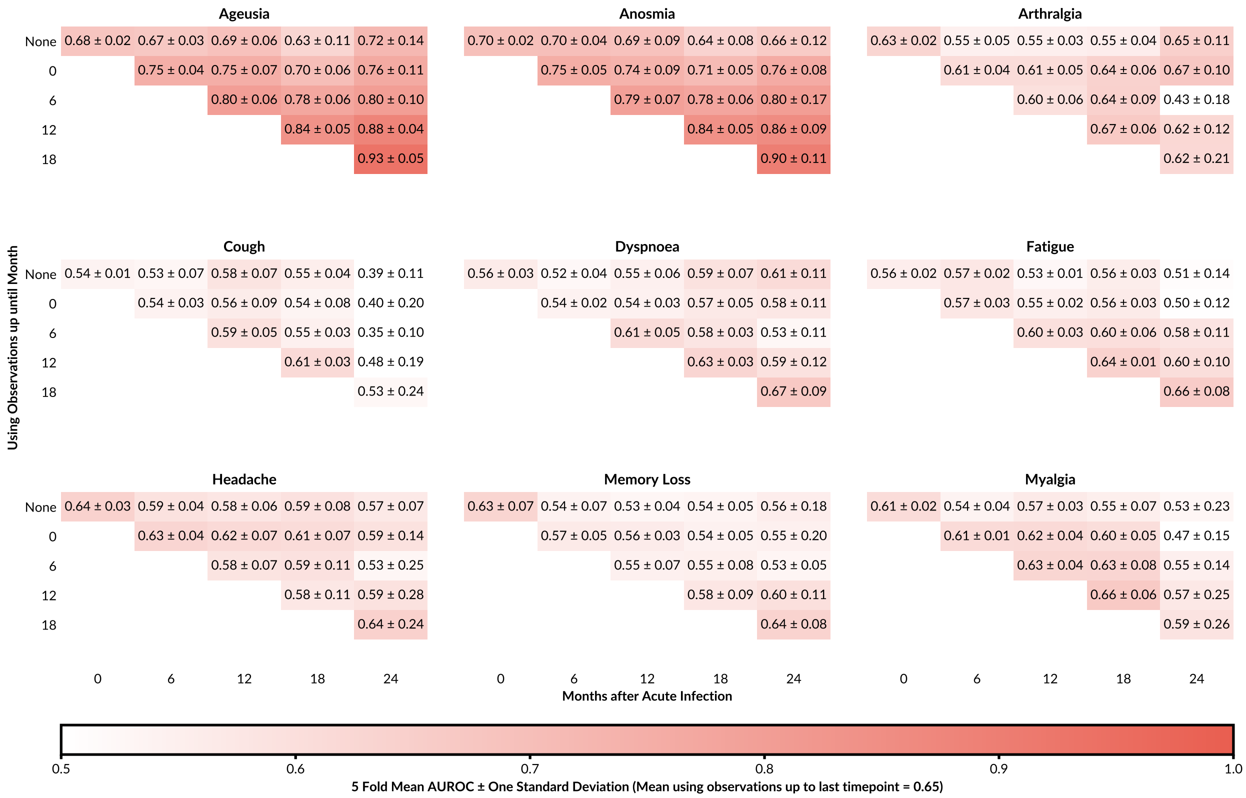

Supplementary Figure 9: **Cross Validation of AUROC and Mean Log Values.** 5-Fold Cross Validation Results of AUROC and Mean Log Values of Symptoms and SF-36 Scores respectively.

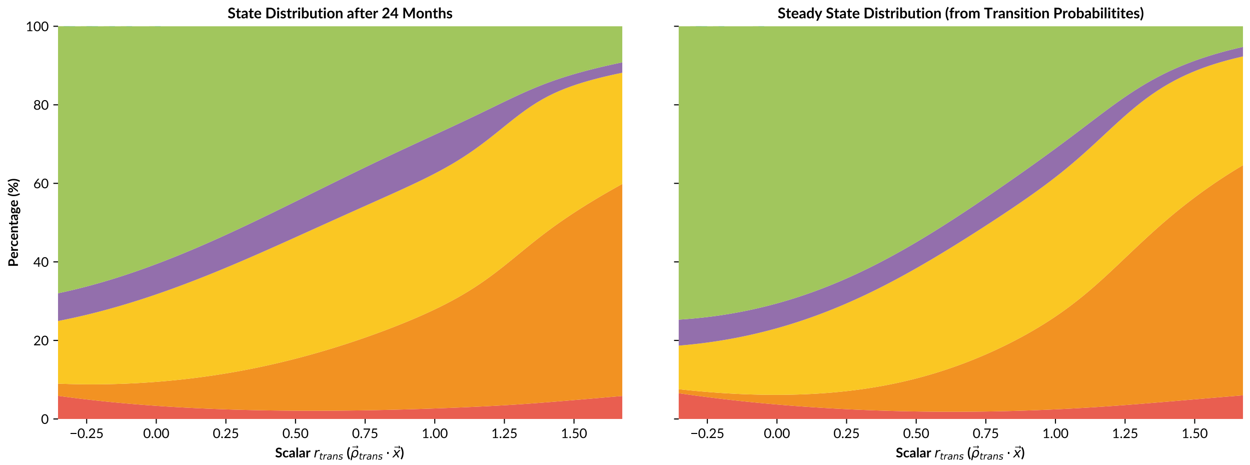

Supplementary Figure 10: **Steady State Distribution Comparison.** Comparing 24 Months and Steady State Distributions for varying r^{trans}.

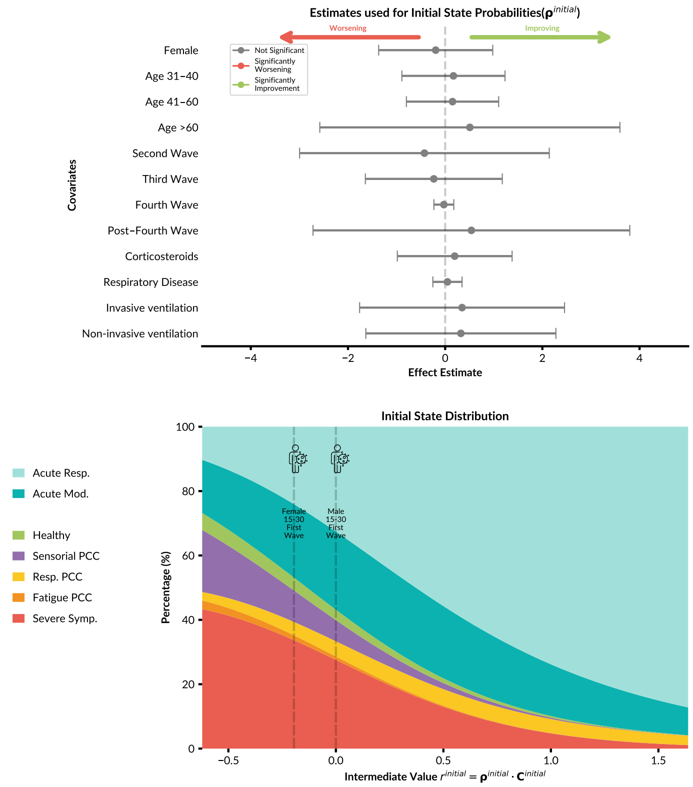

Supplementary Figure 11: **Acute Phase Covariate Impact and Initial State Distribution.** Acute phase covariates impact on the initial probability of states. A Wald test was performed for each parameter where all p-values were two-sided. No adjustment for multiple comparisons was applied. The 95% confidence bands were computed from the parameter variance–covariance matrix.
