# Supplementary material for: Latent transition analysis for longitudinal studies of post-acute infection syndromes
Source: Nat Commun. 2026 Feb 10;17:2557. doi: 10.1038/s41467-026-68650-7 (PMC13000239; doi:10.1038/s41467-026-68650-7)
Supplement: Supplementary file 2 — Description of Additional Supplementary Files [file 41467_2026_68650_MOESM2_ESM.pdf]

Description of Additional Supplementary Files

Supplementary Data 1: Patient Characteristic Summaries across Timepoints. Patient summary statistics of the ORCHESTRA patient population across time of initial infection and follow-up timepoints.

Supplementary Data 2: Symptom Frequency Summaries across Timepoints. Summary of symptoms frequencies of the ORCHESTRA patient population across time of initial infection and follow-up time points. Missing patients are those that had a follow-up but the symptom was not recorded.

Supplementary Data 3: SF-36 Physical and Mental HRQoL Scores across Timepoints. Summary of SF-36 physical and mental QoL questionairre measurement of the ORCHESTRA patient population across time of initial infection and follow-up timepoints.
