# Supplementary material for: Latent transition analysis for longitudinal studies of post-acute infection syndromes
Source: Nat Commun. 2026 Feb 10;17:2557. doi: 10.1038/s41467-026-68650-7 (PMC13000239; doi:10.1038/s41467-026-68650-7)
Supplement: Supplementary file 6 — Reporting Summary [file 41467_2026_68650_MOESM6_ESM.pdf]

nature portfolio

Corresponding author(s): Prof. Jan Hasenauer

Last updated by author(s): 3-12-2025

Reporting Summary

Nature Portfolio wishes to improve the reproducibility of the work that we publish. This form provides structure for consistency and transparency in reporting. For further information on Nature Portfolio policies, see our Editorial Policies and the Editorial Policy Checklist.

Statistics

For all statistical analyses, confirm that the following items are present in the figure legend, table legend, main text, or Methods section.

n/a	Confirmed	
☐	☒	The exact sample size (*n*) for each experimental group/condition, given as a discrete number and unit of measurement
☐	☒	A statement on whether measurements were taken from distinct samples or whether the same sample was measured repeatedly
☐	☒	The statistical test(s) used AND whether they are one- or two-sided<br>*Only common tests should be described solely by name; describe more complex techniques in the Methods section.*
☐	☒	A description of all covariates tested
☒	☐	A description of any assumptions or corrections, such as tests of normality and adjustment for multiple comparisons
☐	☒	A full description of the statistical parameters including central tendency (e.g. means) or other basic estimates (e.g. regression coefficient) AND variation (e.g. standard deviation) or associated estimates of uncertainty (e.g. confidence intervals)
☒	☐	For null hypothesis testing, the test statistic (e.g. *F*, *t*, *r*) with confidence intervals, effect sizes, degrees of freedom and *P* value noted<br>*Give P values as exact values whenever suitable.*
☒	☐	For Bayesian analysis, information on the choice of priors and Markov chain Monte Carlo settings
☒	☐	For hierarchical and complex designs, identification of the appropriate level for tests and full reporting of outcomes
☒	☐	Estimates of effect sizes (e.g. Cohen's *d*, Pearson's *r*), indicating how they were calculated

Our web collection on statistics for biologists contains articles on many of the points above.

Software and code

Policy information about availability of computer code

Data collection	Study data were collected and managed using the REDCap electronic data capture tool (Research Electronic Data CAPture).
Data analysis	- R 4.3.3 was primarily used for the preprocessing of the dataset.<br>- All data formatting for input into the model, simulation, model implementation, model selection, performance evaluation and exportation of results were conducted in Julia version 1.10.1. The implementation can be found online on https://doi.org/10.5281/zenodo.17787061.<br>- Plotted figures were created in Python 3.10.12.

For manuscripts utilizing custom algorithms or software that are central to the research but not yet described in published literature, software must be made available to editors and reviewers. We strongly encourage code deposition in a community repository (e.g. GitHub). See the Nature Portfolio guidelines for submitting code & software for further information.

Data

Policy information about availability of data

All manuscripts must include a data availability statement. This statement should provide the following information, where applicable:

- Accession codes, unique identifiers, or web links for publicly available datasets
- A description of any restrictions on data availability
- For clinical datasets or third party data, please ensure that the statement adheres to our policy

The complete ORCHESTRA WP2 Long Term Sequalea dataset is not openly available due to the sensitivity of the data, but can be requested via an online form

specifying the cohort data of interest, the variables, and the research question to pursue at https://dataportal.orchestra-cohort.eu/data_access/. It is possible to directly access a subset of anonymized data via the public use file at https://dataportal.orchestra-cohort.eu/public_use_file

Research involving human participants, their data, or biological material

Policy information about studies with human participants or human data. See also policy information about sex, gender (identity/presentation), and sexual orientation and race, ethnicity and racism.

Reporting on sex and gender

Sex was included in the analysis as a relevant covariate.

Reporting on race, ethnicity, or other socially relevant groupings

Ethnicity was collected but not included in the analysis.

Population characteristics

COVID-19 confirmed patients over 14 years old with laboratory-confirmed SARS-CoV-2 infection and written informed consent. At the acute infection time point, there were:

First wave: 2591 patients (50.9%)
Second wave: 725 (14.2%)
Third wave: 881 (17.3%)
Fourth wave: 481 (9.4%)
After fourth wave: 416 (8.2%)

At least one vaccination dose before acute infection: 543 (10.7%)

No Admission to Hospital: 1254 (24.6%)
General Hospital Admission: 2757 (54.1%)
ICU Hospital Admission: 1067 (20.9%)

For more detailed patient summaries, see Supplementary Table 7.

Recruitment

Specific strategies for enrollment were site-specific. Enrollment was mainly conducted during medical evaluation for COVID-19 (e.g. hospitalization, evaluation for early antiviral therapy such as anti-spike monoclonal antibodies); enrollment was possible also during clinical evaluation for Long COVID. Most of the patients were enrolled before vaccination. The strategy included more patients with severe and critical COVID-19 than the general population of COVID-19 patients, with an increased risk of Post-COVID-19 condition (PCC), also known as Long COVID. Due to the above-mentioned recruitment strategy, estimates for the overall risk of PCC are subject to being biased in this analysis.

Ethics oversight

University of Verona Ethical Committee approved on 13th April 2021

University of Bologna Ethical Committee approved on 3rd June 2021

French Covid, Institut National de la Santé et de la Recherche Médicale Ethical Committee approved on 7th February 2020

Andalusian Health Service Ethical Committee approved on 19th March 2020

COVID-HOME, University Medical Center Groningen Ethical Committee approved on 28th May 2021

Note that full information on the approval of the study protocol must also be provided in the manuscript.

Field-specific reporting

Please select the one below that is the best fit for your research. If you are not sure, read the appropriate sections before making your selection.

☒ Life sciences ☐ Behavioural & social sciences ☐ Ecological, evolutionary & environmental sciences

For a reference copy of the document with all sections, see nature.com/documents/nr-reporting-summary-flat.pdf

Life sciences study design

All studies must disclose on these points even when the disclosure is negative.

Sample size

Not applicable. Long COVID was unknown at the time of protocol writing. As an observational study, we tried to maximize enrollment to define as much as possible Long COVID. The sample size is of sufficient size to mitigate concerns of overfitting, support reliable estimation of model parameters and to allow for generalizable conclusions from the fitted model.

Data exclusions

For this analysis, only patients who completed at least 1 follow-up visit after COVID-19 were included.

Replication

The assessment of the models with 4 to 8 latent states revealed a high degree of structural similarity (Supplementary Figure 3), as well as similar covariate impact (Supplementary Figure 4), which demonstrates the consistency of the results reported. To evaluate model

performance of the final 7 state model, the chosen set of covariates was used in a 5 fold cross validation procedure with 20 multistarts, each with a cold starting parameter vector.

Randomization	Not applicable. Observational study.
Blinding	Not applicable. Observational study.

Reporting for specific materials, systems and methods

We require information from authors about some types of materials, experimental systems and methods used in many studies. Here, indicate whether each material, system or method listed is relevant to your study. If you are not sure if a list item applies to your research, read the appropriate section before selecting a response.

Materials & experimental systems

n/a	Involved in the study
☒	☐ Antibodies
☒	☐ Eukaryotic cell lines
☒	☐ Palaeontology and archaeology
☒	☐ Animals and other organisms
☐	☒ Clinical data
☒	☐ Dual use research of concern
☒	☐ Plants

Methods

n/a	Involved in the study
☒	☐ ChIP-seq
☒	☐ Flow cytometry
☒	☐ MRI-based neuroimaging

Clinical data

Policy information about clinical studies

All manuscripts should comply with the ICMJE guidelines for publication of clinical research and a completed CONSORT checklist must be included with all submissions.

Clinical trial registration	NCT05097677
Study protocol	https://www.clinicaltrials.gov/study/NCT05097677
Data collection	Data were collected during acute phase (SARS-CoV-2 infection) and during follow-up at 6, 12, 18 and 24 months. The follow-up visits were conducted in dedicated clinics. As this project includes protocols that started during the first wave of COVID-19, the first patient enrolled dates to February 2020 (France). The last follow-up visit has been performed in May 2024.<br><br>Congo and Argentina were initially included as recruiting centers, but failed to share data accordingly to GDPR.
Outcomes	This analysis includes clinical assessments of COVID-19 Sequelae. Other analyses are ongoing.<br><br>The presence of PCC was defined in accordance with WHO definition for PCC (reference provided in the manuscript), which rely on “clinically unexplained symptoms”. Symptom presence was confirmed by an in-person visit with a registered clinician, and symptoms explained by another known cause were discarded (e.g., dry eyes in Sjögren’s disease). Quality of life measures were evaluated through a validated questionnaire, the SF-36, using official translations for local languages.

Plants

Seed stocks	*Report on the source of all seed stocks or other plant material used. If applicable, state the seed stock centre and catalogue number. If plant specimens were collected from the field, describe the collection location, date and sampling procedures.*
Novel plant genotypes	*Describe the methods by which all novel plant genotypes were produced. This includes those generated by transgenic approaches, gene editing, chemical/radiation-based mutagenesis and hybridization. For transgenic lines, describe the transformation method, the number of independent lines analyzed and the generation upon which experiments were performed. For gene-edited lines, describe the editor used, the endogenous sequence targeted for editing, the targeting guide RNA sequence (if applicable) and how the editor was applied.*
Authentication	*Describe any authentication procedures for each seed stock used or novel genotype generated. Describe any experiments used to assess the effect of a mutation and, where applicable, how potential secondary effects (e.g. second site T-DNA insertions, mosiacism, off-target gene editing) were examined.*
